# Inoculating a New Generation: Immunology in Medical Education

**DOI:** 10.3389/fimmu.2019.02548

**Published:** 2019-11-01

**Authors:** Constantine G. Haidaris, John G. Frelinger

**Affiliations:** ^1^Department of Microbiology and Immunology, University of Rochester School of Medicine and Dentistry, Rochester, NY, United States; ^2^The Wilmot Cancer Center, University of Rochester School of Medicine and Dentistry, Rochester, NY, United States

**Keywords:** immunology, medical, education, digital, clinical

## Abstract

Educating the next generation of physicians is a key means of communicating and disseminating impactful immunologic scientific knowledge, and its practical application to human disease. We present our perspective, using as our model a first-year medical school course entitled Host Defense. As the name suggests, immunology is the overarching principle that links the multiple subjects in the course. We address a range of immunologically relevant topics, including innate and adaptive immunity, vaccines, inflammation, allergy, tumor immunotherapy, transplantation, and autoimmunity. These topics are integrated with the fields of infectious diseases, pathology, clinical laboratory testing, and public health, to illustrate how the basic science discoveries in immunology are relevant to clinical practice. The course objectives are not only to deliver “first principles” and molecular mechanisms, but also to connect these principles with the clinical world of diagnosis and therapy. We detail the different methodologies used to achieve these objectives and to reach today's medical students. This provides a framework for course structure and execution designed to engage both the novice and the more “immunologically experienced” learner. The framework includes classical didactic components and personalized instructor access, aligned with current approaches to self-directed learning and using digital media. We also address some of the challenges of assembling a course like Host Defense in the context of an academic medical center with multiple scientific, educational, and clinical missions. This perspective is not meant be proscriptive, but rather to outline our experiences on the strategies tried, while describing their advantages and drawbacks in teaching immunology.

## Introduction

Connecting the concepts of immunology to the clinic is a challenge for medical students (1, 2). To quote a clinician/educator at our institution, “Of all the science topics covered in medical school, immunology was one of the hardest to wrap my head around.” Achieving this goal is not trivial for either learner or instructor. The learner can be daunted by the ever expanding “alphabet soup” constituting the language of immunology; cytokines, chemokines, effector molecules, cell types, cell surface receptors. The instructor cannot elucidate immunology's basic concepts without extensive use of terminology. Furthermore, effective teaching of immunological concepts requires integration of basic knowledge from multiple disciplines in the context of clinical observations and laboratory findings (3, 4).

We describe strategies for teaching immunology to first year medical students. In a course entitled Host Defense, we integrate immunological topics with the fields of infectious diseases, pathology and laboratory testing to explore the impact of the immune response on human health. The course is designed to deliver “first principles,” and to connect these principles with the clinical world of diagnosis and therapy. Herein, we address the following questions:

- What are the main challenges of course organization?
- How can we integrate digital media into education?
- How does one connect basic science to the clinical world in a way that is both educational and meaningful?
- What are some emerging trends in immunology education?

In this Perspective, we describe strategies that worked well for us, and some that did not. We also provide specific examples in the hope that others might adapt these strategies in their unique medical education and immunology teaching settings.

## Logistics

Logistics, “the detailed coordination of a complex operation involving many people, facilities, or supplies” is an underappreciated, yet crucial, part of any course. The importance of logistics has long been appreciated by the military.

“Amateurs talk about tactics, but professionals study logistics.” –Gen Robert H. Barrow, USMC, as well as others.

“My logisticians are a humorless lot … they know if my campaign fails, they are the first ones I will slay.” –Alexander the Great.

Running a course that involves multiple lecturers, spans several disciplines and includes activities outside of lecture, presents a logistical challenge. Importantly, from the faculty standpoint, the quote from Alexander is relevant—if there are problems with delivery of the material, or performance of medical students on standardized tests such as USMLE Step 1, it is the director of the course who pays the price! Thus, the logistics of the course matters; determining the number of hours of didactic instruction, organizing specific topics to optimize the flow of ideas, scheduling, and recruiting lecturers as well as facilitators for Problem Based Learning (PBL) groups are just some of the hurdles.

We must now also grapple with the challenge of integrating the digital world into a course in ways that engage students, provides current, accurate information, and enhances learning. Table 1 summarizes the advantages and disadvantages of e-resources we utilized in Host Defense. To bridge the traditional and digital worlds, we advocate a hybrid strategy where selected content can be delivered using a self-study, electronic format (5), so that in-person lecture can be used to integrate key concepts in the context of health and disease (6). A full description of our course structure, learning objectives and lecture materials is provided in Table S1. The next several sections describe our experiences, and challenges we faced in organizing the course and its content.

**Table 1:** Activities and resources used in host defense: advantages and disadvantages.

**Activity/Modality**	**Advantages**	**Drawback/Disadvantage**
In-class demonstrations by presenter using props and models.	Potential to engage and involve students: serve as a “memory peg” for learning. Provides a break allowing students to re-focus.	Students may remember the demonstration but not the immunological concept. Time consuming.
Small group exercises in class. Pose a question and discuss.	Enhances peer-to-peer engagement. Presenter can quickly assess if students are progressing and can discuss answers in real time.	Time consuming. Instructor must keep a relatively firm hand on organization or it can become chaotic.
iPad for content delivery.	Ability to store large amounts of information, searchable, can annotate files, and look things up in real time. Can view textbooks, slides, and lecture notes in class.	May be a distraction; e.g., shopping, messaging with friends. Annotating notes can detract from classroom awareness.
E-flash cards for vocabulary.	Self-directed and self-paced learning. Will accommodate images, audio, and video links and text. Can provide pre-made cards or have students build their own sets.	Preparation is work-intensive. Only a portion of the class may use them. If you select one specific application, it can become obsolete and/or unpopular.
Audience e-response tools.	Rapid feedback to students. Increases student engagement. Can quickly determine if they are absorbing concept.	Must commit to the technique and the specific tool. If system falters, student attention quickly diminishes.
iBooks for teaching clinical laboratory.	Provides opportunities for interactivity not available in a PDF format.	Work-intensive to assemble. Once assembled, cumbersome to edit.
Case studies in infectious diseases sponsored by the Infectious Diseases Society of America.	Clinical cases compiled by experts in infectious diseases and presented in an interactive, expository format. Many cases annotated for medical students.	Not an encyclopedic collection, but growing. Found at idimages.org.
Interactive white board application for iPad.	Fosters collaborative interaction in real time in digital realm. Useful as a study tool for a group and to generate interactive “mind maps.”	Slow response time of Wi-Fi network, and alternative personal preferences, led to its rapid demise.
Visual Dx.com	Electronic dermatology image database of an extensive array of diseases, with examples across the range of human skin pigmentation. Addresses lack of diversity.	Institutional access requires a subscription.
Twitter peer-to-peer and student-to-faculty communication	Followed by entire class in real time. Can easily retweet relevant articles linked to breaking immunology topics. Many students use Twitter.	Need to use consistently, can only use for certain tasks; limited by length of content; requires some digital skill.

## Transition to Digital Content

Many features of digital content delivery appeal to today's students. Links embedded in a document, and the ability to look up unfamiliar terms or find digital images instantly, enrich the learning experience (7). These advantages led many medical schools, including our own in 2012, to use electronic tablets (in our case, iPads) to deliver didactic content. Using electronic content freed us from printing a 650-page syllabus weeks in advance, allowing editing of the content closer to lecture. Over time, we moved from syllabus replacement to using the iPad to deliver new material linked to lecture content. With the invaluable help of our institution's instructional design expert, we introduced on-line modules to explore diagnostic laboratory microbiology (8, 9). Modules on bacteriology and virology were contained in five iBooks linked to clinical cases (www.idimages.org), each followed by a computer-based self-assessment of knowledge related to the diagnostic tests (Table 1).

We did face challenges in using iPads for content delivery. To quote Marshall McLuhan, “The medium is the message” (10). We found there were unanticipated consequences to introducing new technology that changes the inter-personal dynamics between instructor and learner. During lecture, students focusing on the iPad, and not the lecturer, detracted from the ability of the lecturer to “read” the audience and gauge the effectiveness of their delivery. Unfortunately, this parallels the filing of electronic medical records while interviewing a patient, to the dismay of patients and physicians alike. The interaction between the student and the lecturer is further compromised if the student succumbs to the temptation to use the tablet or laptop for activities unrelated to lecture, e.g., shopping, messaging with friends, etc., as their attention wanders (11).

## The Perils and Pitfalls of E-Learning Tools

While the iBooks used to explore diagnostic microbiology were viewed favorably, we cite two experiences where introducing electronic learning tools into Host Defense did not proceed as smoothly as hoped.

Learning vocabulary remains an essential step in immunology and, indeed, all of medicine. Clinicians use this vocabulary daily, and remark that medical vocabulary is the major part of the first 2 years of medical school. Although many students view memorization of terminology pejoratively, there is no more rapid means to shred professional credibility than to mangle the vocabulary. Defaulting to “However you say it …” is no longer acceptable.

In consultation with both students and our instructional design team, we prepared an extensive set of e-flashcards with application *Study*© for the vocabulary of immunology and infectious diseases to be used in a self-study format. The application was purchased by our institution, provided to each student and formal instruction offered. Along with text, incorporating audio allowed us to add the proper pronunciation for a given term. Despite expending considerable effort to create the e-flashcards, it did not translate into widespread utilization by students. One colleague quipped, “If *you* build it, they *won't* come.” Course surveys revealed only ~25% of the class found the e-flashcards “very useful.” In contrast, a professional, visually based program employing “memory pegs” (12) and animation, *SketchyMicro*©, was considerably more popular, with ~75% finding it “very useful.” Illustrating the gap between students and faculty, we were initially unaware of the degree to which *SketchyMicro*© was adopted, even though the more popular application was relatively expensive and available only for rent.

In a second instance, we observed students using an interactive computer whiteboard to share content in real time over the Internet and create concept maps (13, 14) as a study tool. With the help of these students and our instructional design expert, we introduced and demonstrated a free, interactive whiteboard iPad application to the entire class during a lecture. We tasked all students to use the application in their respective Problem Based Learning (PBL) group to replace the conventional classroom whiteboard. Our goal was to make it easier to share learning objectives and concept maps of Host Defense PBL cases with the class. Disappointingly, our “top-down” approach quickly crashed, and the students stopped using it after 1–2 sessions. Students stated that the response time of the network was too slow to keep pace with the group's discussion. It was faster to simply write on the board and take a picture on their phone. Furthermore, many students had already been using other platforms, such as Google Docs, and were unwilling to switch. We learned the hard lesson that students often outpace faculty in identifying and adopting new digital applications. Moreover, their popularity can change rapidly through peer-to-peer communication to which faculty are often not privy.

## Copyright and Fair Use

The advent of digital content delivery raises the question: How does one use textbooks, particularly in the context of lectures? Does one create all one's own figures (a time consuming and daunting task) or use existing material? In the latter case, there are numerous immunology textbooks, with excellent, professionally designed figures. However, with the steady decline in the purchase of textbooks by students, copyright issues rise to the fore.

Issues surrounding Fair Use of copyrighted material depend upon the precise circumstances when they are used (15–18). Instructors have long used published figures to supplement their lectures, and this has generally been deemed permissible. However, if the course materials are posted on-line, ease of re-distribution can pose copyright problems. If the library buys a site license for a course text, this issue can be mitigated to some extent. However, as we have experienced, if the library buys a site license and later discontinues it, you may need to redo the digital content for your entire course. Posting class materials on portals with access restricted to registered students, such as Blackboard, may serve as an important barrier to potential copyright infringement. Nevertheless, if copyright infringement is alleged, the instructors are usually left to fend for themselves (15). Your institution's library staff is a good resource for Fair Use guidelines to help navigate these issues.

## Making Connections Between Disciplines

**Figure 1:**
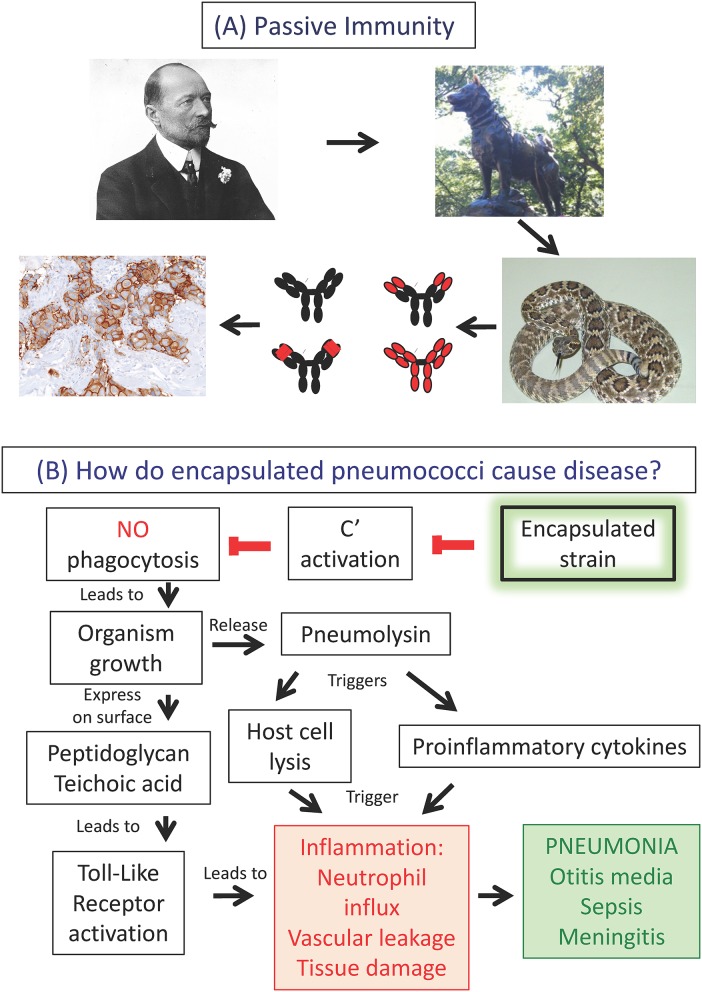
Making connections between disciplines. **(A)** Antibodies in immunity, disease and therapy. **(A)** Illustrates one example used in didactic lectures to make connections in immunology. This slide design is used in lecture to create “memory pegs” between material covered in the course and to demonstrate how many of the same basic principles can be applied to several clinically relevant situations. Here we show a picture of the Nobel prize winner von Behring who developed diphtheria antitoxin. This form of passive immunity was memorably applied in the delivery of antitoxin by the sled dog Balto and his owner Gunnar Kaasen for the treatment of an outbreak of diphtheria in Nome, Alaska. From here one can segue into the role of antibodies in treating snakebites, the structure of antibodies to minimize immune complex disease, the modern use of passive immunization using humanized monoclonal antibodies such as Herceptin® (trastuzumab) for tumor immunotherapy, and other related topics such as Rh disease. Links to additional slides and other educational resources for teaching Immunology can be found at the American Association of Immunologists (AAI) website (https://www.aai.org/Education/Teaching-Resources). **(B)** Connecting infectious disease, inflammation and adaptive immunity with concept mapping using the bacterium *Streptococcus pneumoniae*, the pneumococcus, as an example. How do encapsulated pneumococci cause disease? Inhaled encapsulated strains fail to activate complement, thereby evading phagocytosis by alveolar macrophages followed by outgrowth of the organism. Bacterial cell walls, containing peptidoglycan and teichoic acid, activate Toll-like receptors, inducing inflammation. Concomitantly, the bacterium releases the protein pneumolysin, lysing lung cells and inducing proinflammatory cytokines, thereby exacerbating inflammation. Neutrophil influx, vascular leakage and tissue damage manifest as pneumonia, with potential dissemination of infection to extra-pulmonary sites.

Understanding immunological concepts requires the expert integration of multiple disciplines and concepts. Can you teach someone to be an expert in immunology in a medical school class? Obviously, not; becoming an expert takes years of intense effort and dedication. However, it is possible to illustrate how experts *think* by using examples to make connections between topics students perceive as disparate (19). To illustrate passive immunity, we described the delivery of anti-toxin by the sled dog Balto for the treatment of an outbreak of diphtheria in the Inuit population of Nome, Alaska (20). This was used to segue into serum sickness, Lupus, Rh disease, rattlesnake bite therapy, monoclonal antibodies, and tumor immunotherapy (Figure 1A). We have also used concept mapping (13, 14) to connect the fields of infectious disease, inflammation and adaptive immunity. In a lecture “From Bacterial Capsules to Vaccines,” we start with classic studies from the 1920's on infection caused by *Streptococcus pneumoniae* to describe how a bacterial structure, polysaccharide capsule, results in evasion of phagocytosis, leading to lung inflammation and consequent pneumonia (Figure 1B). We then transition to the bacterial capsule as an immunogen to explore the concepts of antibodies as opsonins, pneumococcal serotypes, conjugate vaccine design, and immune evasion using the same concept mapping approach.

## Connecting to the Clinic

Medical students recall immunological concepts most effectively when they are placed in a clinical context (6, 21–23). We make clinical correlations by incorporating cutting-edge immunology topics in the news and examining mishaps that occur in medicine, such as transplanting a mismatched kidney. We can review not only the immunology involved, but also encourage discussions on medical ethics. To strengthen the link between basic and clinical immunology (24), we conduct in-class small group exercises to measuring immune responses, with emphasis on the uses of antibodies. Further, we have a series of PBL sessions based on clinical cases emphasizing basic science that, with the help of a facilitator, students work through as a team.

We incorporate physicians into the course; as lecturers in their area of expertise to connect basic science to clinical care, but also to communicate how they think about patients (25). We have a clinical immunologist deliver lectures on inflammation, hypersensitivity, asthma, and autoimmunity, and the roles of monoclonal antibodies and other biologics in the therapy of immunologic diseases. We then reinforce and expand these concepts by recruiting a dermatologist to lecture on cutaneous manifestations of adverse drug reactions. We also have two in-class sessions on clinical decision-making in infectious diseases. Clinicians describe their own cases and the decisions they made in terms of diagnosis, therapy and follow-up; emphasizing the evolution of their thinking over time. While clinical vignettes can never fully replicate the experience of a physician connecting with an individual patient for whom they are responsible, they can demonstrate how an expert physician integrates basic science into clinical medicine. The physicians inter-weave all aspects of patient history with basic and clinical science, while communicating their sense of responsibility for the patient's well-being.

The clinical lectures by physicians also serve as an important bridge between the basic science and clinical spheres, and illustrate how basic science information is applied. For example, as shown in Figure 1, we explore the role of the antibody and complement in promoting phagocytosis of encapsulated bacteria. Complement and immune complexes are reintroduced in the context of serum sickness resulting from the passive immunization against diphtheria toxin using horse serum (see Balto, Figure 1), and later in the context of immune complex diseases such as systemic lupus erythematosus (SLE). Complement comes up yet again in a discussion of immunodeficiencies, exemplified by increased susceptibility to infection by the bacterium *Neisseria meningitidis*, as well as increased frequency of autoimmune diseases. The spaced repetition of the complement system in different contexts is not only an excellent learning tool (26), but also helps to integrate the multidisciplinary field of immunology.

Integrating clinicians into a course poses challenges. First, the lecturer often over-estimates the students' clinical knowledge. Consequently, students often feel overwhelmed by their presentations. It is also hard to schedule clinicians to fit within the flow of the course, as their patient care responsibilities always come first. Finally, the clinicians usually do not have the time to examine the course content in detail. A common expression uttered, which never fails to cause considerable consternation among students (and the directing faculty), is “I don't know if you've had this yet, but…,” giving the unfortunate perception that the course is disorganized and lecturers do not communicate with each other. We coach the lecturers to not use that phrase (not always successfully) by emphasizing where we are in the lecture series and the relative level of audience expertise.

## Offering the Best of Both Worlds

We embrace the utility of digital resources and understand their appeal (see Table 1 for details of resources and activities used in Host Defense, along with pros and cons). However, we feel strongly that the most important component of our course is a traditional one; direct interaction with students, in person. Students frequently request that lectures be video recorded; this is problematic from several standpoints. Viewing a video of a good lecture cannot adequately replace the dynamic of *attending* a good lecture, with the opportunity to view the spectrum of instructor-student interactions, questions, and comments. Video recording of lectures also inevitably leads to a decrease in attendance, resulting in less interaction with instructors and with peers (27).

Effective interaction with a large class requires moving beyond standing at the podium, holding forth for an hour and then exiting the room. We use several approaches to facilitate that interaction, summarized in Table 1. For example, to restore waning student attention during lectures, students are routinely called upon to participate in demonstrations in front of the class that illustrate major teaching points. We also intersperse lectures with small group activities to both make teaching points and help foster teamwork. The instruction team must also find a balance between course objectives and the time students need to master the material. We provide in-class time to perform computer-based exercises to provide personalized instruction, if needed. The course director attends all lectures, and is available to consult with students in the lecture hall when no formal lectures are scheduled, a time we have termed Questions and Answers (Q and A).

Like many institutions, we use similar multiple choice questions to those on Step 1 USMLE as one of our assessment mechanisms. However, it is challenging to construct questions that truly assess students' grasp of conceptual knowledge or their ability to synthesize and apply concepts in immunology. To address this issue, we have tried several types of writing exercises that also provide feedback to instructors as to gaps in the student's knowledge. Our current approach, favored by students and instructors alike, is a small-group exercise performed outside of class explaining the underlying immunology involved in an article or video from a popular media source. This reflected the increasing frequency of immunology-based treatments, or clinical scenarios involving immunology, described in commercial or social media, with the expectation that their future patients will want explanations of these new treatments. The group could either choose an article or pick from a list provided. For example, one article titled “HIV used to cure ‘bubble boy' disease” instead described using gene therapy to cure severe combined immunodeficiency disease. Each group was tasked with explaining the immunologic mechanisms of the treatment, its advantages over previous approaches, potential drawbacks, or adverse consequences, cost considerations, and any biomedical errors perceived in the article. Their report was limited to two pages, including a picture or diagram of the immunologic mechanisms involved and a description of the issues just described. All students were expected to read the reports of the other groups. Students valued the opportunity to be creative, work as a team, and to take an active role in directing their learning process.

In all these exercises, logistics, in terms of planning, timing in the lecture and smooth execution, are critical. Faculty time, commitment and direct in-person guidance are essential to maintain their organization and assure communication of the outcomes of the activities to the entire class. Since the initiation of significant course re-modeling in 2012, student surveys demonstrated an increase in the quality of teaching and the quality of the course overall. We used a Likert-like rating scale from 1 to 5 with the following categories: 1. “Needs much improvement,” 2. “Needs some improvement,” 3. “Satisfactory,” 4. “Good” and 5. “Excellent.” Ratings for the course overall improved steadily from slightly below “Satisfactory” in 2011, with an average score of 2.80, to scores consistently in the “Good” to “Excellent” category in 2015 through 2018, with averages ranging from 4.29 to 4.41. Concomitantly, the ratings for overall quality of teaching in 2015–2018 were also in the “Good” to “Excellent” category, with averages ranging from 4.36 to 4.47.

## Discussion

We advocate a self-study, electronic format to deliver specific content (5) that affords lecture time to integrate key concepts in the context of health and disease (6). Appreciating that learning may be enhanced by complementing didactic lectures with interactive activities (2, 7, 28, 29), lecture can be supplemented with brief, small group activities during lecture, and in more detailed PBL sessions spanning several days. This hybrid approach is extremely flexible. Recognizing that digital technologies and innovations are constantly being developed, one can blend and experiment with digital advances, while maintaining the best of traditional methods.

The experiences we have described are with medical education in the U.S. We have also utilized the hybrid approach in our basic science courses in microbiology and immunology for undergraduates and graduate students. Moreover, we believe that these lessons will also be valuable to educators outside of the U.S. because many of the challenges faced, particularly on how to incorporate the ever-expanding modes of delivering information, are shared concerns. These educational issues include the balance of traditional methods such as lectures with electronic resources, the increasing adoption and preferences of students for digital modalities, and the role of broad electronic platforms such as internet web sites and social media. These issues are common to educational endeavors wherever one teaches. This shared experience is reflected in studies from outside the U.S. cited herein, including those on student interest in immunology (Australia) (1), use of electronic tablets (United Kingdom) (3) and e-resources (Brazil, Germany, Switzerland) (2, 28, 29) in teaching, and connecting basic science to the clinical world (Canada) (3, 23, 25). Furthermore, it is increasingly recognized that educational strategies must be developed for teaching immunology in the resource-constrained regions of the developing world (30). Open access to internet-based, digital resources (2), such as those listed in Faggioni et al. (31), will facilitate closing the gaps between under-served regions and developed areas of the world. In addition, through their respective Education Committees, the International Union of Immunological Societies (iuisonline.org, in association with immunopaedia.org) and the American Association of Immunologists (aai.org/Education/Teaching-Resources) are committed to providing and disseminating quality digital educational resources, as well as organizing meetings and courses, to fill this need. We hope that the strategies we propose herein will help guide the use of these electronic resources effectively.

Looking to the future, we see three emerging technological trends that we anticipate will make major impacts in teaching immunology and related disciplines. They include:

1. A multi-institution collaboration to develop a “shared medical school curricular ecosystem” has been proposed (32, 33) using online videos to deliver core content to preclinical students, thereby affording faculty more class time to facilitate personalized, interactive learning experiences.
2. The increased incorporation of social media (34) including blogging (35) and Twitter (36–38), to facilitate student-student and student-faculty communication.
3. The integrated analysis of the human immune response and systems immunology (39), which require concomitant development of both basic immunological literacy and information literacy skills (40–42) early in medical training.

Whatever the future holds, one can be certain that Immunology will impact nearly every aspect of a physician's practice (24). The sophisticated technological approaches that will become “normal” for today's students as they move into medical practice will be deprived of their potential promise without fostering life-long learning and interest in immunology early in their training. However, we are cognizant of a time-tested quote:

“The only thing constant is change” –Heraclitus.

In that light, we advocate a blend of methods to teach the concepts and applications of immunology, but one that affords the flexibility to adapt to changing times. Immunologists, of course, excel at adapting!

## Author Contributions

CH and JF contributed equally to the concept, organization and writing of this manuscript.

### Conflict of Interest

The authors declare that the research was conducted in the absence of any commercial or financial relationships that could be construed as a potential conflict of interest.
