# Supplementary material for: Inoculating a New Generation: Immunology in Medical Education
Source: Front Immunol. 2019 Nov 1;10:2548. doi: 10.3389/fimmu.2019.02548 (PMC6843008; doi:10.3389/fimmu.2019.02548)
Supplement: Supplementary file 1 [file Table_1.DOCX]

**Table S1. Course Logistics**

| **Course Structure** | Length | 7 weeks; 4 hr per day Mon.-Fri. Includes lectures, class time to complete on line modules, Problem Based Learning sessions, Question and Answer sessions, exams. |
| --- | --- | --- |
|  | Class Composition | 104 first year undergraduate medical students. |
|  | Teaching Faculty | Five Basic Sciences faculty, four Clinical faculty. |
|  | Lectures, including in-class demonstrations and small group activities | 67 hr; 32 hr infectious diseases, 31 hr immunity and host-pathogen interactions, 4 hr integrating basic science principles with clinical decision-making. |
|  | Large Group | 22 hr; “Questions and Answers” with Course Director and other teaching faculty. |
|  | Problem Based Learning (PBL) | 24 hr; 4 cases, 2 hr/day, Mon., Wed., Fri. 12 groups of 8-9 students each, with one Facilitator per PBL group. |
|  | On-Line Modules | 17 x 1 hr each; Laboratory Diagnostic Techniques, Case Studies in infectious diseases and immunity. |
|  | Exams | 3 x 3 hr each, multiple choice and short answer questions based on course and individual lecture Learning Objectives, along with a Small Group Activity requiring approx. 6 hr outside class. |
| **Course Learning Objectives** | ***Infectious Diseases***   1. *Recognize a presentation of infectious disease and suggest approaches to identify the most likely causative agent.* 2. *Describe the mechanisms used by infectious agents to cause pathology.* 3. *Describe the physical nature of microorganisms and how this determines the options for appropriate antimicrobial therapy.* | When presented with a patient history of respiratory, genitourinary, gastrointestinal, bone, skin, or neurological infection, list the most likely etiologic agents among the bacteria, viruses, fungi, or parasites known to cause disease. Suggest diagnostic tests to differentiate among the possibilities.  When presented with a specific microorganism, describe the specific aspects of the organism that allow it to cause disease in the host. In addition, describe how the host response to infection can contribute to pathology.  Describe the structural differences among bacteria, viruses, fungi & parasites, and how these differences dictate the selection of chemotherapy. List the major classes of anti-infective agents and their respective modes of action. Describe the mechanisms used by infectious agents to thwart the efficacy of chemotherapy. |
|  | ***Innate and Adaptive Immunity***   1. *Describe the cellular and acellular components associated with innate immunity and their function.* 2. *Describe the series of events that results in the generation of humoral and cellular immunity.* 3. *Explain the concept of self and non-self as it relates to transplantation.* 4. *Identify, distinguish, and suggest treatment among the types of hypersensitivity.* 5. *Explain the strategies for vaccine development.* 6. *Apply the fundamentals of immunology to new strategies for the prevention and treatment of disease.* | Describe the physical and chemical barriers, cell types, the key receptor-ligand interactions, and the key effector molecules (complement, anti-microbial peptides and lipids, interferons) associated with innate immunity. Explain how these components interact with microorganisms to control infection and stimulate the adaptive immune response.  Explain the process of formation of specific antibody to its cognate antigen. This should include a consideration of antigen processing and presentation, T-cell stimulation of B-cell activation, the structure of immunoglobulin isotypes, antibody affinity versus avidity, and the function of the different domains of antibodies in antigen recognition, complement activation and phagocytosis. Describe the genetic mechanisms of antibody isotype switching and the generation of antibody diversity.  Explain how the immune cells are educated to distinguish between self and non-self, including the concepts of thymic education, histocompatibility antigens, and cytotoxic T cells.  When presented with a case of hypersensitivity, distinguish among the 4 types by considering time to reaction, clinical presentation, and experiential history of patient. The student should be able to name the immune cells and molecules involved in the reaction, and propose possible treatments.  Determine the type of immunity required for protection against individual pathogens for each major class of infectious agent. Describe how to identify appropriate vaccine targets for an infectious agent. Describe how to determine the predicted efficacy of a vaccine by evaluating the appropriate correlates of protection in immunized individuals. Describe the role of B and T cells in the development of a protective immune response to the vaccine target.  Connect basic principles in immunology to new developments in immunotherapy; including the use of monoclonal antibodies for the treatment of cancer and allergy, the development of viral vectors for gene therapy, and immunological approaches to limit the impact of bioterrorism. |
| **Lecture Materials** | Lecture Slides | Provided as a PDF in advance of lecture. |
|  | Lecture Notes | Each set includes a list of 4-7 specific Learning Objectives. Notes are designed to complement, not duplicate, the Lecture Slides. Provided as a PDF in advance of lecture. |
